# Dietary organic acid supplementation improved productive performance, jejunal morphology, immune status, antioxidant- and inflammation-related gene expression, blood biochemistry, and meat quality of broiler chickens

**DOI:** 10.1016/j.vas.2026.100866

**Published:** 2026-09-11

**Authors:** Hassan Shirzadi, Enayat Rahmatnejad, Hassan Habibi

**Affiliations:** a Department of Animal Science, Faculty of Agriculture, Ilam University, Ilam, Iran; b Department of Animal Science, Faculty of Agriculture and Natural Resources, Persian Gulf University, Bushehr, 75169, Iran

**Keywords:** Antioxidant status, Broiler chicken, Feed efficiency, Immune response, Meat quality, Organic acids

## Abstract

This experiment investigated the influence of dietary inclusion of an organic acid blend (OAB) on productive performance, carcass characteristics, jejunal morphology, immune responses, antioxidant- and inflammation-related gene expression, blood biochemistry, and meat quality in broilers over a 42-day trial. 120 1-day-old chicks were randomly allocated to either a basal diet or the same diet supplemented with 0.1% OAB. OAB supplementation decreased body weight gain (BWG) and feed intake (FI) during the starter period but increased BWG and European production efficiency index (EPEI) and improved feed conversion ratio (FCR) during the finisher period. Over the entire experimental period, OAB improved FCR and tended to increase EPEI (P = 0.096), whereas BWG and FI were unaffected. At day 42, OAB increased jejunal villus height and villus surface area. OAB supplementation decreased the heterophil percentage and heterophil-to-lymphocyte ratio, enhanced the jejunal expression of NFE2L2 and SOD1, and reduced TNFα expression. Birds fed OAB also showed higher serum glucose, total protein, and albumin concentrations. OAB reduced press loss and cooking loss in breast muscle and press loss in thigh muscle, increased the pH of both muscles, and tended to reduce cooking loss in thigh muscle (P = 0.091). These findings demonstrate that dietary inclusion of OAB enhances feed efficiency, intestinal development, antioxidant and immune functions, and meat quality attributes in broilers.

## Highlights

• Organic acid blend (OAB) improved finisher growth and feed efficiency.
• OAB increased jejunal villus height and villus surface area.
• OAB upregulated NFE2L2 and SOD1 and reduced TNFα expression.
• OAB increased serum glucose, total protein, and albumin levels.
• OAB improved meat quality by reducing water losses and increasing pH.

## Graphical abstract

**Figure:**
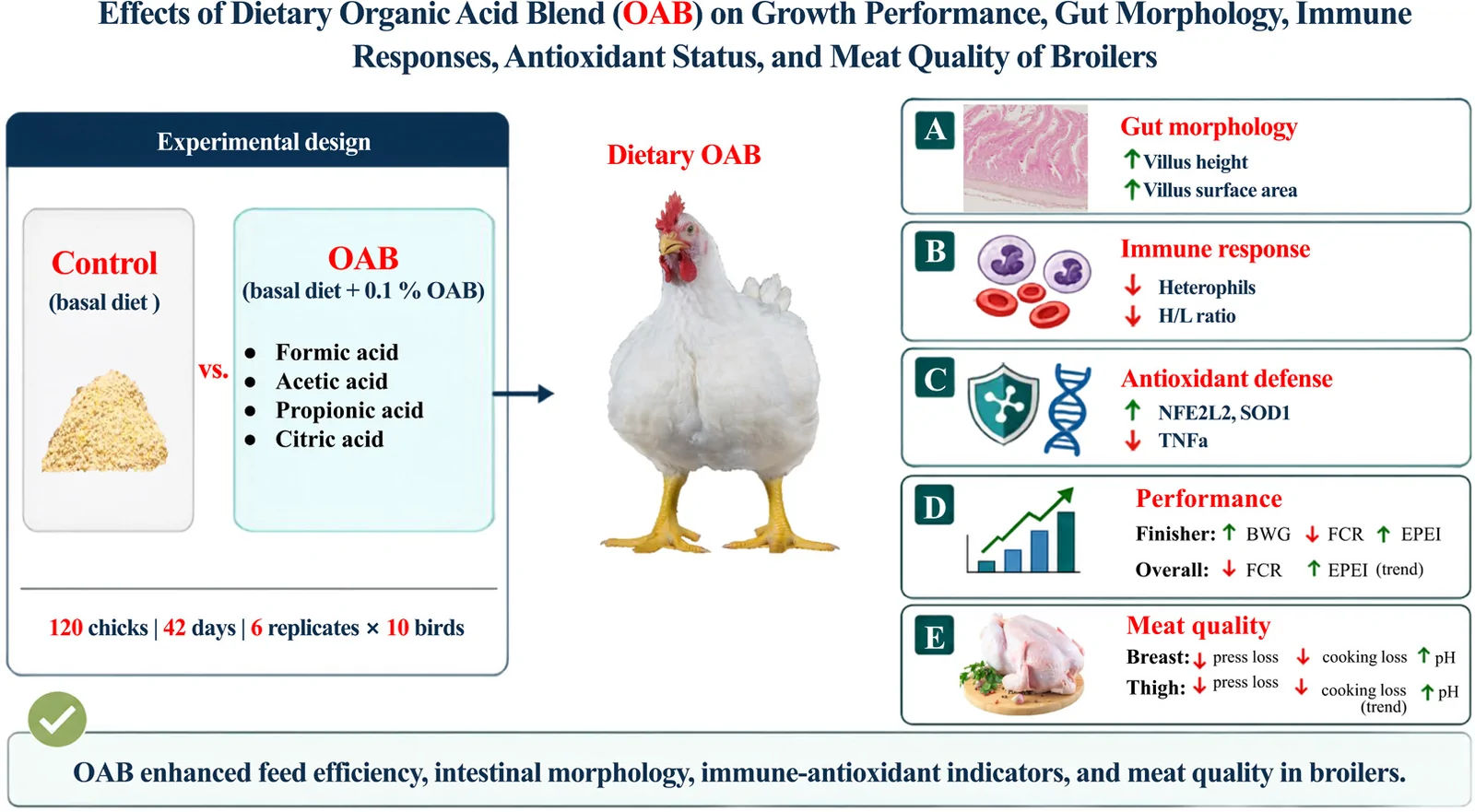

## 1 Introduction

The routine use of antibiotic in poultry nutrition has been increasingly restricted worldwide because of concerns over the emergence of antimicrobial-resistant bacteria and the presence of antibiotic residues in poultry-derived foods (Castanon, 2007; Du et al., 2024; Gadde et al., 2017; Yang et al., 2025). Consequently, considerable research attention has focused on nutritional strategies capable of maintaining bird health and production efficiency without relying on antibiotic growth promoters. Several nutritional alternatives to antibiotics have been explored, including probiotics and prebiotics (Shehata et al., 2022; Yang et al., 2025), phytogenics (Khairunnesa et al., 2026; Khatun et al., 2026), organic acids (Du et al., 2024; Khan et al., 2022), exogenous enzymes (Ibrahim et al., 2023), and other functional feed additives. Among these alternatives, organic acids have attracted particular interest because of their potential effects on gastrointestinal function, microbial balance, nutrient utilization, and productive performance.

Organic acids are weak carboxylic acids that may be administered individually or as blends through feed or drinking water. Based on their chemical structures, they can be broadly classified as monocarboxylic acids or hydroxycarboxylic acids (Dibner & Buttin, 2002). Increasing evidence indicates that blends of different organic acids often produce greater biological responses than individual acids because they combine complementary modes of action and exert antimicrobial activity throughout gut (Dibner & Buttin, 2002; Polycarpo et al., 2017; Samanta et al., 2010). Previous studies have consistently shown that the dietary inclusion of organic acids enhances growth performance (Abdelli et al., 2020; Islam et al., 2024), gut health (Emili Vinolya et al., 2021), gut microbiota (Islam et al., 2024; Rodjan et al., 2018), and immune response (Houshmand et al., 2012).

These beneficial responses are generally explained by several complementary mechanisms. Organic acids reduce gastrointestinal pH, thereby facilitating the enzymatic conversion of pepsinogen into pepsin, improving protein digestion, increasing nutrient utilization, and enhancing mineral availability (Dibner & Buttin, 2002; Park et al., 2009). In addition, they suppress pathogenic microorganisms by lowering luminal pH and allowing undissociated acid molecules to penetrate microbial cell membranes, ultimately disrupting intracellular homeostasis (Scicutella et al., 2021). Furthermore, short-chain fatty acids (SCFA) are utilized as an energy substrate by intestinal epithelial cells, stimulate epithelial renewal, reinforce barrier integrity, and contribute to the maintenance of intestinal homeostasis (Liu et al., 2021).

Despite extensive research on growth performance and intestinal health, the molecular and physiological responses of broilers to dietary organic acid blends remain incompletely understood. In particular, limited information is available regarding their effects on the intestinal expression of antioxidant- and inflammation-related genes and whether these responses occur concurrently with changes in immune status, blood biochemistry, and meat quality. Simultaneous evaluation of these outcomes could provide a more integrated understanding of the biological responses to organic acid supplementation and clarify whether improvements in productive performance are accompanied by changes in intestinal, physiological, and meat-quality traits.

Therefore, the present study evaluated the effects of dietary supplementation with an organic acid blend (OAB) containing citric, acetic, formic, and propionic acids on productive performance, carcass characteristics, jejunal morphology, immune responses, jejunal expression of antioxidant- and inflammation-related genes, blood biochemical indices, liver enzyme activities, and meat quality in broiler chickens. We hypothesized that OAB supplementation would improve productive and intestinal morphology and induce favorable molecular, physiological, and meat-quality responses.

## 2 Materials and methods

### 2.1 Experimental materials

The OAB evaluated in this study was Vivacid® (Vivan Co., Mashhad, Iran), a commercial preparation containing a combination of citric, acetic, formic, and propionic acids.

### 2.2 Study procedures and bird management

In a 42-day trial, 120 one-day-old Ross 308 broilers weighing 41 ± 0.8 g were used. Birds were distributed randomly into two dietary treatments, each comprising six replicate cages with 10 chicks per replicate. The experiment comprised two dietary treatments: a control group receiving the basal diet and a treatment group fed the basal diet supplemented with 0.1% OAB.

Birds were housed in wire-floored cages. Each cage was equipped with feeder and drinkers. Feed and water were provided ad libitum throughout the experiment. The basal diets (Table 1) were formulated according to the nutrient specifications of the Ross 308 broiler management guidelines for the starter (0–10 d), grower (11–24 d), and finisher (25–42 d) phases. The birds were maintained in an environmentally controlled facility. The ambient temperature was initially maintained at 33 °C and was gradually reduced to 22 °C by day 27, at which level it was maintained until the end of the experiment. Relative humidity was maintained at approximately 50%. Based on these environmental conditions, the temperature-humidity index (THI), calculated according to Dedousi et al. (2023), averaged 25.5 during the first 27 days and 20.8 from day 28 to day 42 (Supplementary Figure S1). The lighting program consisted of 23 h light: 1 h dark until day 7, followed by 20 h light: 4 h dark from days 8–42. To enable the evaluation of humoral immune responses, all birds were vaccinated against Newcastle disease and avian influenza on day 14. No other vaccines or therapeutic medications were administered during the experimental period to avoid potential interference with the antibody titers measured on day 28.

**Table 1:** Composition and calculated analysis of the basal diet (%, unless otherwise stated).

**Item**	**Starter** (0–10 d)	**Grower** (11–24 d)	**Finisher** (25–42 d)
**Ingredients**			
Corn grain	55.10	59.79	65.21
Soybean meal (44%)	32.39	27.96	24.17
Corn gluten meal	5.19	5.76	4.48
Corn oil	1.87	1.91	1.92
L-Lysine	0.48	0.42	0.41
DL-Methionine	0.39	0.33	0.32
L-Threonine	0.19	0.15	0.14
L-Arginine	0.17	0.15	0.16
L-Valine	0.09	0.05	0.07
Premix^1^	0.50	0.50	0.50
DCP	2.11	1.69	1.38
Limestone	0.89	0.66	0.62
Sodium Bicarbonate	0.37	0.35	0.34
Common Salt	0.16	0.18	0.18
Sand^2^	0.10	0.10	0.10
**Calculated analysis**			
ME (Kcal/kg)	2975	3050	3100
CP	23.00	21.50	19.5
Linoleic acid	1.34	1.43	1.53
Crude fiber	3.55	3.35	3.18
Calcium	0.95	0.75	0.65
Available phosphorus	0.50	0.42	0.36
Sodium	0.18	0.18	0.18
Potassium	0.83	0.76	0.70
Chlorine	0.23	0.23	0.23
DCAB^3^ (mEq/kg)	226.72	208.01	191.57
SID^4^ lysine	1.32	1.18	1.08
SID methionine	0.71	0.64	0.60
SID methionine + cystine	1.00	0.92	0.86
SID threonine	0.88	0.79	0.72
SID tryptophan	0.21	0.19	0.17
SID arginine	1.40	1.27	1.17
SID leucine	1.87	1.82	1.65
SID isoleucine	0.83	0.77	0.69
SID valine	1.00	0.91	0.84

1 Supplied per kilogram diet: vitamin A, 9000 IU; vitamin D3, 2000 IU; vitamin E, 18 IU; vitamin K3, 2 mg; riboflavin, 6.6 mg; pantothenic acid, 10 mg; pyridoxine, 3 mg; folic acid, 1 mg; thiamin, 1.8 mg; B12, 15 µg; biotin, 0.1 mg; niacin, 30 mg; choline, 500 mg; Se, 0.2 mg; I, 1 mg; Cu, 10 mg; Fe, 50 mg; Zn, 85 mg; Mn, 100 mg.

2 The experimental diet was formulated by substituting 0.1% sand in the basal diet with 0.1% organic acid blend (OAB; Vivacid®).

3 Dietary cation-anion balance.

4 Standardized ileal digestible.

### 2.3 Productive performance and carcass traits

Body weight and feed intake (FI) were recorded on days 0, 10, 24, and 42 of the experiment. Body weight gain (BWG) and feed conversion ratio (FCR) were subsequently determined for each experimental period and for the overall trial. Mortality was monitored daily for inclusion in performance calculations, but none occurred during the experimental period. In addition, the European production efficiency index (EPEI) was calculated using the following equation (Shirzadi et al., 2026; Usturoi et al., 2023):

EPEI=[Livability×Finalbodyweight]/[Age×FCR]×100

On days 28 and 42, two birds from each replicate whose body weights were closest to the replicate average were selected for sampling. Blood was collected from the wing vein before birds were humanely euthanized for carcass evaluation. The relative weights of the carcass, breast muscle, leg (thigh and drumstick), heart, liver, bursa of Fabricius, spleen, and thymus were calculated as a percentage of live body weight. The same birds were subsequently used for analyses of jejunal morphology, immune responses, jejunal expression of immune- and antioxidant-related genes, serum biochemical variables, liver enzyme activities, and meat quality and chemical composition.

### 2.4 Jejunal morphology

Samples were collected from the midpoint of the jejunum on days 28 and 42 immediately after euthanasia. Jejunum specimens were immersed in 10% neutral-buffered formalin, dehydrated and cleared using routine histological procedures, embedded in paraffin and sectioning into 6-μm-thick slices with a rotary microtome. The prepared sections were then stained with hematoxylin and eosin (H&E) and examined histologically. Morphometric evaluation was performed using a light microscope. Height (VH) and width (VW) of villus and crypt of depth (CD) were measured, after which the villus height-to-crypt depth ratio (VH:CD) and villus surface area (VSA) were calculated (Thuekeaw et al., 2022):

### 2.5 Immune responses

On day 28, blood samples were obtained via venipuncture of the wing vein from the selected birds and separated into two aliquots. One portion was used to prepare blood smears, whereas the other was centrifuged to obtain serum. After air-drying, the blood smears were stained with Wright–Giemsa stain for differential leukocyte counting. Differential leukocyte counts were performed under a light microscope by counting 100 leukocytes per smear, and the percentages of heterophils (H) and lymphocytes (L) were recorded. Then the heterophil-to-lymphocyte ratio (H:L) was calculated. Serum samples were stored at −20 °C in sterile microtubes until determination of antibody responses. Antibody titers against avian influenza virus (AIV) and Newcastle disease virus (NDV) were determined using the hemagglutination inhibition (HI) test following the procedure described by Sedaghat and Torshizi (2017).

Cell-mediated immune responsiveness was evaluated by measuring cutaneous basophil hypersensitivity following challenge with 2,4-dinitro-1-chlorobenzene (DNCB) and phytohemagglutinin-P (PHA-P). Skin thickness was measured 24 h after DNCB application to a featherless area and at 24 and 48 h after intradermal injection of PHA-P into the interdigital skin, as described by Sedaghat and Torshizi (2017).

### 2.6 RNA extraction and quantitative real-time PCR

A segment of the mid-jejunum was collected on day 28 and carefully rinsed with phosphate-buffered saline (PBS) to remove luminal contents, and immediately immersed in RNAlater solution for preservation until laboratory analysis. Equal amounts of jejunal tissue (50 mg) collected from the two sampled birds were pooled before RNA extraction. Each pooled sample was considered one biological replicate, resulting in six biological samples per treatment (n = 6). Total RNA was isolated and its quality was evaluated, followed by cDNA synthesis, primer design and validation, and qPCR analysis, in accordance with the protocol described by Shirzadi et al. (2024). The primer pairs used for qPCR were identical to those reported in our previous study (Karimi Zandi et al., 2026). The expression levels of NF-κB1, TNFα, IL6, IL10, NFE2L2, SOD1, and GPX1 were determined by SYBR Green-based qPCR using a StepOnePlus Real-Time PCR System, with ACTB used as the reference gene.

### 2.7 Blood biochemistry and liver enzymes

Blood samples collected on day 42 were centrifuged to obtain serum for biochemical evaluation. Serum biochemical variables, comprising total protein (TP), albumin, glucose, calcium, phosphorus, and the enzymatic activities of AST, ALT, ALP, and LDH, were analyzed using an automated analyzer for biochemical measurements (BT-1500, Biotechnica, Italy) and commercially available diagnostic kits obtained from Pars Azmoon Co. (Tehran, Iran). Serum globulin concentration was calculated by difference between total protein and albumin.

### 2.8 Meat quality and chemical composition

At 42 days of age, the left breast and thigh muscles were excised for evaluation of meat quality and proximate composition. Meat quality characteristics, including pH (measured 24 h postmortem), drip loss, cooking loss, and press loss, were determined in accordance with the protocol described by Michiels et al. (2012). Proximate composition of the meat, including dry matter, ash, and organic matter, was analyzed following the standard procedures of AOAC (2007).

### 2.9 Statistical analysis

All statistical analyses were conducted using the General Linear Model (GLM) procedure of SAS software (SAS Institute, 2008), based on a completely randomized experimental design. The model used was Yij = μ + Ti + eij, where Yij is the observed response, μ is the mean of treatment, Ti is the effect of dietary treatment, and eij is the error associated with each measurement.

Prior to analysis, the assumptions of normality and homogeneity of variance were verified. All measured variables were analyzed using the cage as the experimental unit. For variables measured in two birds from each replicate cage, the observations were averaged to obtain one value per cage before statistical analysis. Comparisons between the two dietary treatments were performed using Fisher LSD test. Data are expressed as mean ± SEM, and differences were considered statistically significant at P ≤ 0.05, whereas 0.05 < P ≤ 0.10 was interpreted as a statistical tendency.

## 3 Results

### 3.1 Productive performance

**Fig. 1:**
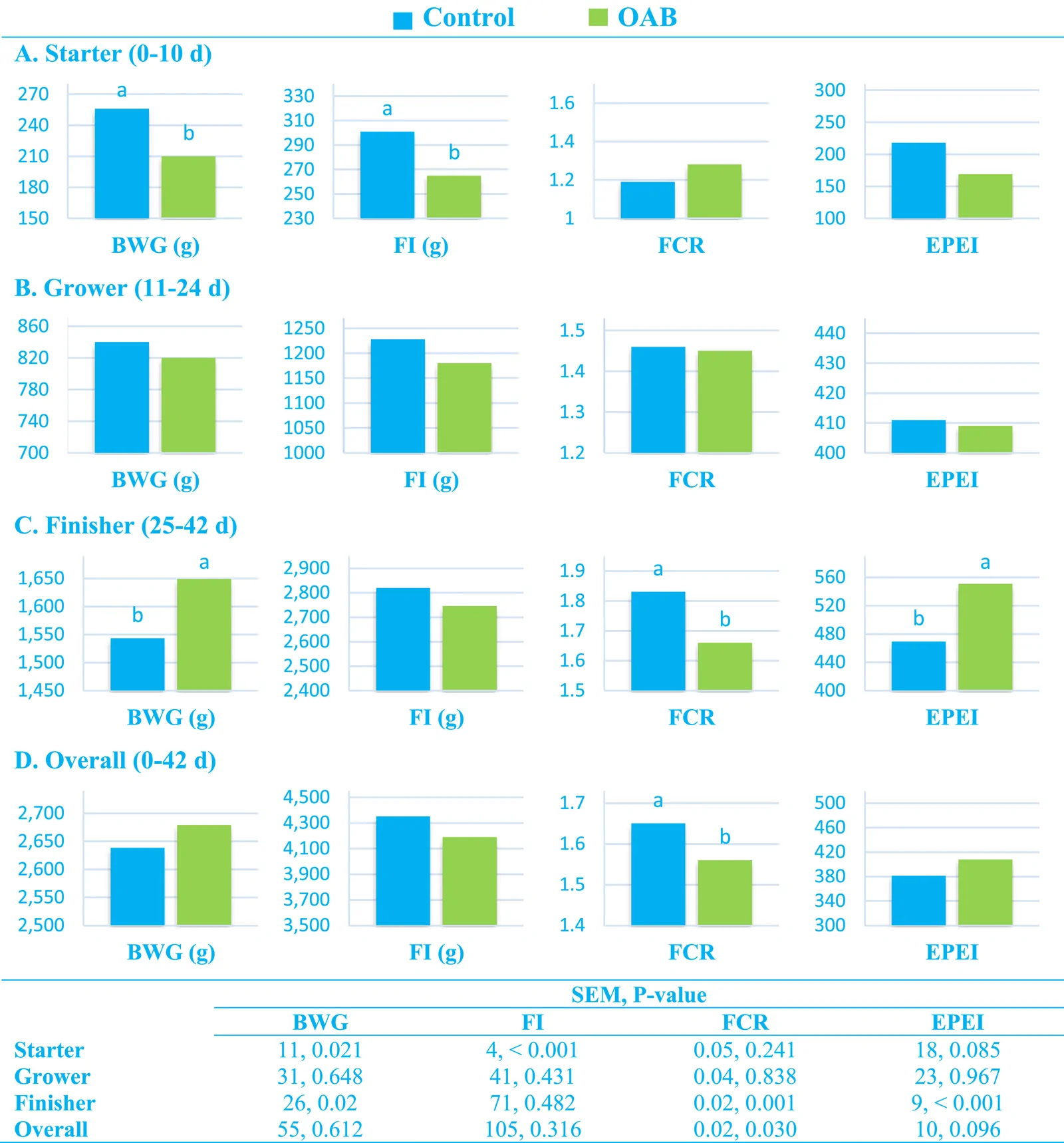
Effects of organic acid on growth performance of broiler chickens. **Control**, birds fed a basal diet; **OAB**, birds fed the basal diet supplemented with 0.1% organic acid blend; **BWG,** body weight Gain; **FI,** feed intake, **FCR**, feed conversion ratio; **EPEI**, European production efficiency index. Bars represent means. ^a-b^ indicates a significant difference between treatments in the same histogram (P ≤ 0.05).

The impact of OAB supplementation on productive performance is illustrated in Fig. 1. During the starter period, OAB supplementation decreased BWG by approximately 18.0% (210 vs. 256 g; P = 0.021) and FI by 12.0% (265 vs. 301 g; P < 0.001) compared with the control. FCR was not affected (P > 0.05), whereas EPEI tended to be lower in OAB-fed birds (P = 0.085). During the grower period, OAB did not affect BWG, FI, FCR, or EPEI (P > 0.05).

During the finisher period, OAB increased BWG by approximately 6.9% (1649 vs. 1543 g; P = 0.020), improved FCR by 9.3% (1.66 vs. 1.83; P = 0.001), and increased EPEI by 17.5% (551 vs. 469; P < 0.001), whereas FI remained unaffected (P > 0.05). Over the entire experimental period, OAB improved FCR by approximately 5.5% (1.56 vs. 1.65; P = 0.030) and tended to increase EPEI (P = 0.096), whereas BWG and FI were unaffected (P > 0.05).

### 3.2 Carcass traits

The effects of OAB on carcass characteristics are shown in Table 2. At either 28 or 42 days of age, no significant differences were observed for carcass yield, breast yield, leg yield, heart, liver, bursa of Fabricius, spleen, or thymus weights at either sampling age (P > 0.05).

**Table 2:** Effects of organic acid on carcass traits of broiler chickens (% of body weight).

**Item**	**Control**	**OAB**	**SEM**	**P- value**
**28 d**				
Carcass	70.4	70.0	1.2	0.809
Breast	22.2	23.5	0.5	0.110
Leg	22.3	22.5	0.9	0.877
Heart	0.637	0.655	0.025	0.619
Liver	3.15	3.23	0.10	0.576
Bursa of Fabricius	0.297	0.252	0.013	0.219
Spleen	0.158	0.160	0.009	0.906
Thymus	0.205	0.189	0.008	0.218
**42 d**				
Carcass	70.9	71.6	1.1	0.667
Breast	24.5	23.9	0.5	0.356
Leg	22.4	23.0	0.6	0.506
Heart	0.717	0.701	0.030	0.712
Liver	2.61	2.74	0.10	0.406
Bursa of Fabricius	0.287	0.268	0.021	0.556
Spleen	0.179	0.128	0.020	0.113
Thymus	0.176	0.204	0.013	0.163

P ≤ 0.05 indicates a significant difference between treatments within each row. **Control**, birds fed a basal diet; **OAB**, birds fed the basal diet supplemented with 0.1% organic acid blend.

### 3.3 Jejunal morphology

The effects of OAB on Jejunal morphology are shown in Table 3. At day 28, OAB supplementation tended to increase VSA (P = 0.078), whereas VH, VW, CD, and VH:CD were unaffected (P > 0.05). At day 42, OAB increased VH and VSA by approximately 10.9% (P = 0.027) and 17.0% (P = 0.046), respectively, and tended to increase VH:CD by 17.3% (P = 0.096). VW and CD were unaffected (P > 0.05).

**Table 3:** Effects of organic acid on jejunal morphology of broiler chickens.

**Item**	**Control**	**OAB**	**SEM**	**P- value**
**28 d**				
VH (µm)	1083	1127	22	0.181
VW (µm)	162	180	10	0.220
CD (µm)	222	204	10	0.250
VH:CD	4.94	5.58	0.34	0.223
VSA (mm^2^)	0.549	0.640	0.032	0.078
**42 d**				
VH (µm)	1150	1275	33	0.027
VW (µm)	186	196	6	0.255
CD (µm)	274	261	11	0.447
VH:CD	4.21	4.94	0.27	0.096
VSA (mm^2^)	0.672	0.786	0.034	0.046

P ≤ 0.05 indicates a significant difference between treatments within each row. **Control**, birds fed a basal diet; **OAB**, birds fed the basal diet supplemented with 0.1% organic acid blend; **VH**, villus height; **VW**, villus width; **CD**, crypt depth; **VH:CD**, the ratio of villus height to crypt depth; **VSA**, villus surface area.

### 3.4 Immune responses

The effects of OAB on immune responses are presented in Table 4. OAB supplementation markedly reduced heterophil percentage and the H:L (P < 0.001). However, dietary OAB supplementation did not influence antibody titers against NDV or AIV (P > 0.05). Similarly, DNCB- and PHA-P-induced responses were unchanged.

**Table 4:** Effects of organic acid on immune responses of broiler chickens.

**Item**	**Control**	**OAB**	**SEM**	**P- value**
Heterophil (%)	34.6	19.4	0.6	< 0.001
Lymphocyte (%)	44.6	46.2	0.9	0.242
H:L	0.776	0.421	0.015	< 0.001
NDV (Log_2_)	5.80	5.40	0.22	0.242
AIV (Log_2_)	3.00	3.20	0.35	0.694
DNCB (mm), 24h	0.241	0.223	0.020	0.551
PHA-P (mm), 24h	0.538	0.620	0.039	0.177
PHA-P (mm), 48h	0.872	0.877	0.017	0.833

P ≤ 0.05 indicates a significant difference between treatments within each row. **Control**, birds fed a basal diet; **OAB**, birds fed the basal diet supplemented with 0.1% organic acid blend; **H:L**, heterophil to lymphocyte ratio; **NDV**, Newcastle disease virus vaccine; **AIV**, avian influenza virus; **DNCB**, 2,4-dinitro 1-chlorobenzene; **PHA-P**, phytohemagglutinin-P.

### 3.5 Expression of immune and antioxidant genes in jejunal tissue

The expression levels of immunity- and antioxidant-related genes are presented in Table 5. Dietary OAB increased the expression of NFE2L2 (P = 0.010) and SOD1 (P < 0.001), and decreased TNFα (P = 0.050), whereas the expression of GPX1, NFKB1, IL6, and IL10 was not significantly affected (P > 0.05).

**Table 5:** Effects of organic acid on jejunal immunity- and antioxidants-related gene expression of broiler chickens.

**Item**	**Control**	**OAB**	**SEM**	**P- value**
NFKB1	1.000	0.973	0.033	0.585
TNFɑ	1.000	0.944	0.017	0.050
IL6	1.000	1.013	0.030	0.762
IL10	1.000	1.059	0.024	0.119
NFE2L2	1.000	1.123	0.026	0.010
SOD1	1.000	1.149	0.006	< 0.001
GPX1	1.000	0.961	0.014	0.088

P ≤ 0.05 indicates a significant difference between treatments within each row. **Control**, birds fed a basal diet; **OAB**, birds fed the basal diet supplemented with 0.1% organic acid blend; **NFKB1**, nuclear factor kappa B subunit 1; **TNFɑ**, tumor necrosis factor alpha; **IL6**, interleukin 6; **IL10**, interleukin 10; **NFE2L2**, nuclear factor erythroid 2 like 2; **SOD1**, superoxide dismutase 1; **GPX1**, glutathione peroxidase 1.

### 3.6 Blood biochemistry and liver enzymes

As shown in Table 6, OAB supplementation increased serum glucose (P = 0.003), total protein (P = 0.039), and albumin concentrations (P = 0.005). No significant effects were observed on globulin, A:G, calcium, phosphorus, AST, ALT, ALP, or LDH activities (P > 0.05).

**Table 6:** Effects of organic acid on blood biochemistry and liver enzymes of broiler chickens.

**Item**	**Control**	**OAB**	**SEM**	**P- value**
Glucose (mg/dl)	219	256	6	0.003
TP (g/dl)	2.48	2.78	0.09	0.039
Albumin (g/dl)	1.20	1.46	0.05	0.005
Globulin (g/dl)	1.28	1.32	0.07	0.710
A:G	0.946	1.133	0.075	0.115
Ca (mg/dl)	8.70	9.36	0.48	0.355
P (mg/dl)	4.24	4.58	0.42	0.351
AST (u/l)	227	215	11	0.450
ALT (u/l)	17.8	16.4	1.1	0.411
ALP (u/l)	3541	3366	137	0.395
LDH (u/l)	1591	1477	89	0.394

P ≤ 0.05 indicates a significant difference between treatments within each row. **Control**, birds fed a basal diet; **OAB**, birds fed the basal diet supplemented with 0.1% organic acid blend; **TP**, total protein; **A:G**, albumin to globulin ratio; **Ca**, calcium; **P**, phosphorus, **AST**, aspartate transaminase; **ALT**, alanine transaminase; **ALP**, alkaline phosphatase; **LDH**, lactate dehydrogenase.

### 3.7 Meat quality and chemical composition

**Fig. 2:**
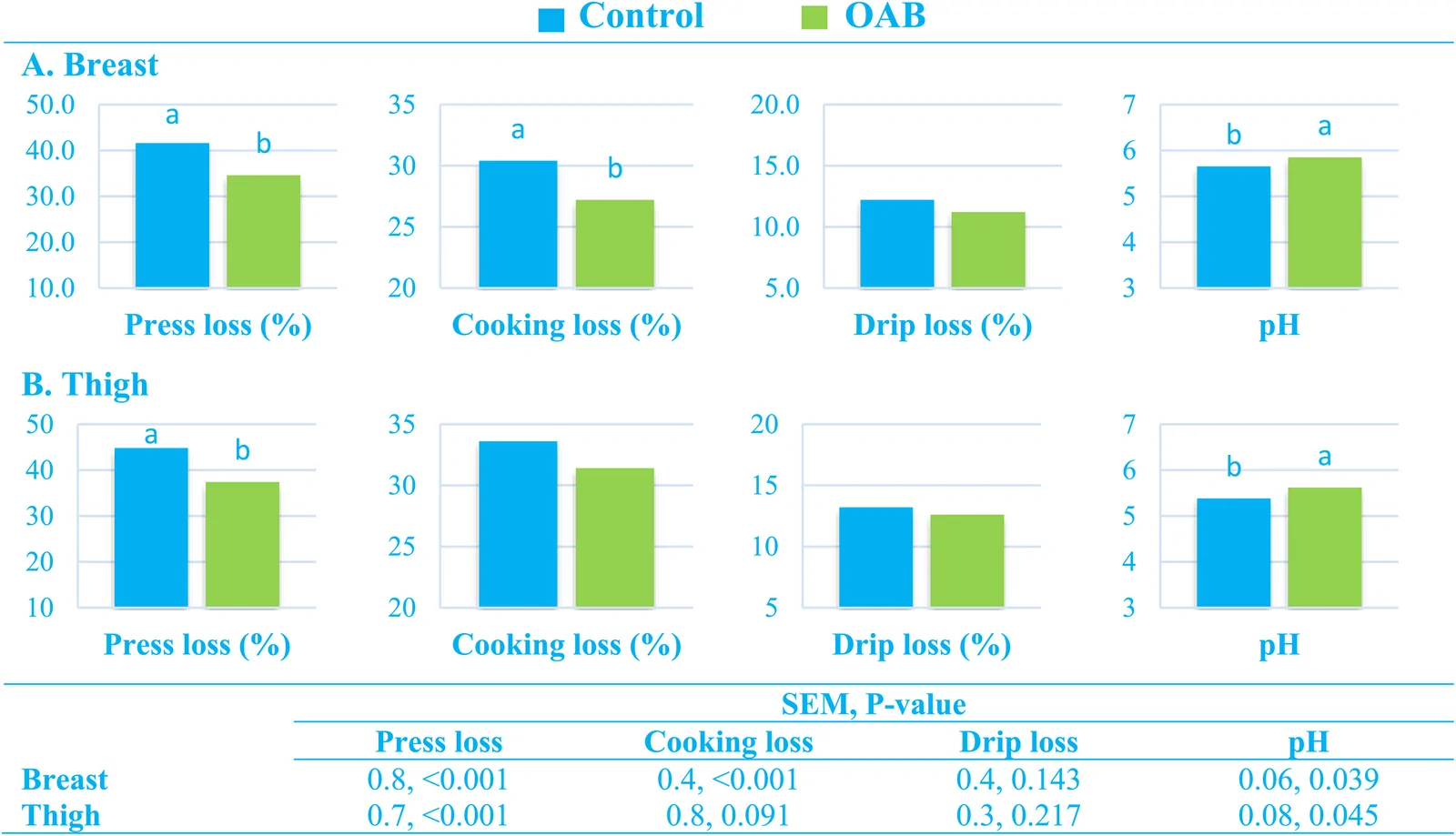
Effects of organic acid on meat quality of broiler chickens. **Control**, birds fed a basal diet; **OAB**, birds fed the basal diet supplemented with 0.1% organic acid blend. Bars represent means. ^a-b^ indicates a significant difference between treatments in the same histogram (P ≤ 0.05).

**Fig. 3:**
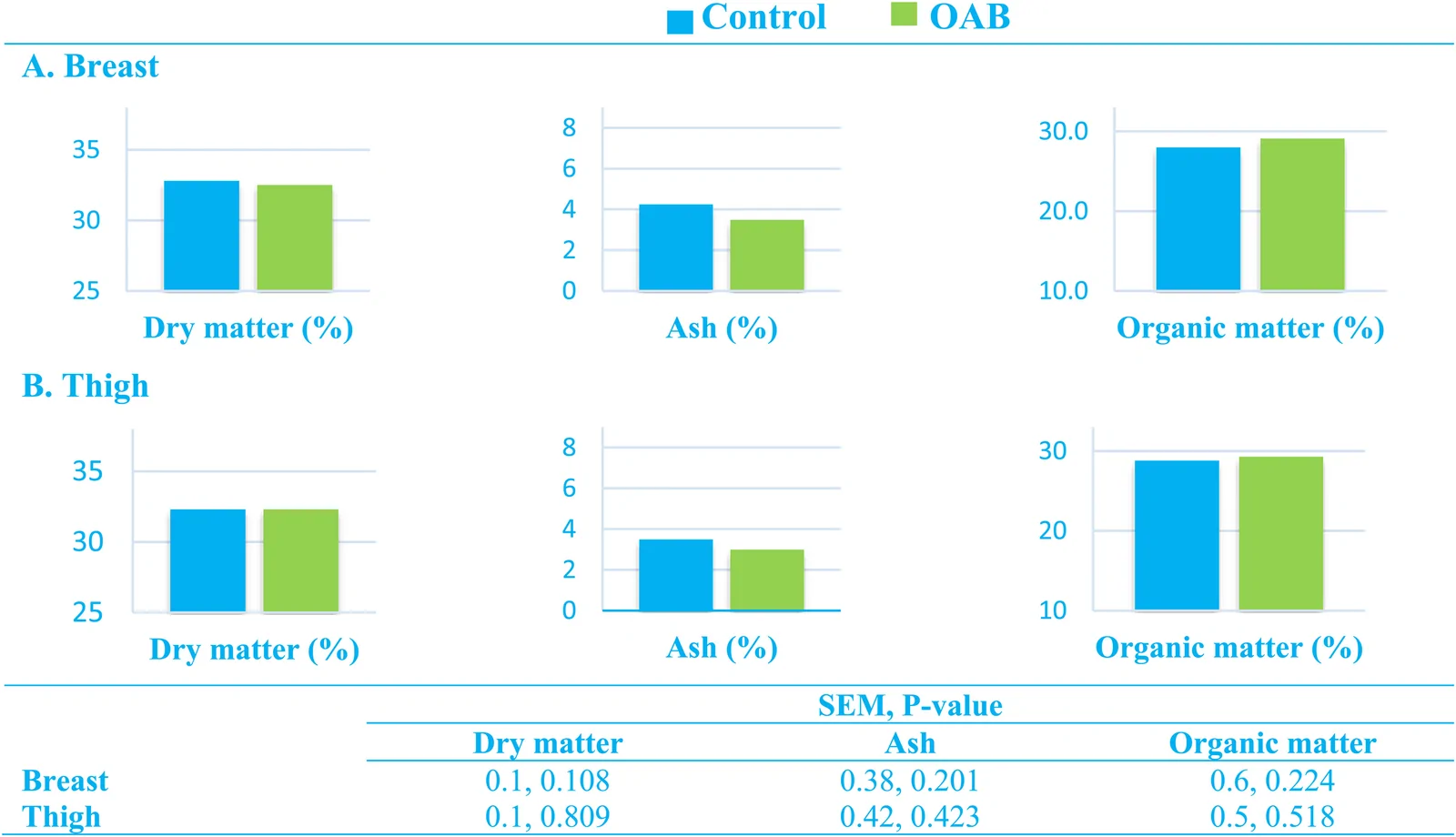
Effects of organic acid on meat composition of broiler chickens. **Control**, birds fed a basal diet; **OAB**, birds fed the basal diet supplemented with 0.1% organic acid blend. Bars represent means. P ≤ 0.05 indicates a significant difference between treatments in the same histogram.

Fig. 2 illustrates the effects of OAB supplementation on meat quality. In breast muscle, OAB significantly reduced both press loss and cooking loss (P < 0.001). Similarly, press loss in thigh muscle was markedly reduced by OAB supplementation (P < 0.001). Although thigh cooking loss was not significantly affected, a tendency toward lower values was observed in OAB-fed birds (P = 0.091). In addition, OAB supplementation increased muscle pH measured 24 h postmortem (P < 0.05), whereas drip loss remained unchanged. As shown in Fig. 3, the chemical composition of breast and thigh muscles, including dry matter, ash, and organic matter contents, was not influenced by dietary OAB supplementation.

## 4 Discussion

This study explored the impact of dietary inclusion of an OAB on productive performance, carcass traits, jejunal morphology, immune function, intestinal expression of genes associated with inflammation and antioxidant defense, serum biochemical parameters, and meat quality attributes of broilers. Although OAB supplementation reduced FI and BWG during the starter phase, it enhanced BWG, FCR, and EPEI during the finisher phase. These improvements resulted in improved overall FCR and a tendency toward improved EPEI.

The transient reductions in FI and BWG observed during the starter phase may reflect a dose- and product-specific response to organic-acid supplementation, as high inclusion levels or the strong taste and odor of some free organic acids can adversely affect feed acceptance and, consequently, early growth responses (Dong et al., 2024). In contrast, coated or microencapsulated formulations allow a more gradual and targeted release of organic acids, potentially reducing their immediate sensory effects and delivering a greater proportion of the active compounds to the distal intestine (van den Borne et al., 2015). Additionally, the observed effect could further be related to the functional immaturity of the gastrointestinal tract during the early post-hatch period (Ravindran & Abdollahi, 2021). Therefore, delayed supplementation, a lower starter-phase inclusion level, or a protected formulation may warrant further investigation as potential strategies to minimize early growth depression. However, these strategies were not directly evaluated in the present study. The present findings agree with those reported by Dong et al. (2024), indicating that dietary OAB supplementation adversely affected growth performance during the starter phase in broiler chickens. Their study demonstrated that providing acidifiers via drinking water resulted in reduced body weight by day 28, together with a decrease in average daily gain over the first 28 days. In contrast, our finding contrasts with studies reporting improved starter-phase performance following organic acid supplementation (Mustafa et al., 2021; Nguyen et al., 2018).

The absence of treatment effects during the grower phase, followed by improved BWG, FCR, and EPEI during the finisher phase, suggests that the response to OAB developed progressively with age. The overall improvement in FCR across the entire trial, despite the absence of a significant effect on overall BWG and FI, further supports the notion that OAB primarily enhances nutrient utilization efficiency rather than FI per se. Organic acids enhance feed efficiency by reducing gastrointestinal pH, triggering the release of various digestive enzymes, increasing nutrient utilization, inhibiting acid-sensitive pathogens, and improving intestinal health through direct actions on epithelial cells, such as providing SCFAs as an energy source for their proliferation (Dibner & Buttin, 2002; Gadde et al., 2017; Nguyen et al., 2018; Yang et al., 2018).

The present findings regarding finisher and overall phases corroborate earlier findings indicating that OAB supplementation enhanced broiler performance (Adil et al., 2010; Dong et al., 2024). Nguyen et al. (2018) indicated the dietary inclusion of medium chain fatty acids and organic acids in broiler chickens linearly increased BWG and improved FCR on day 14 to 35, as well as overall. Responses to organic acids are not always consistent among studies. This discrepancy may be explained by differences in acid blend composition and inclusion level, differences in basal diet composition, buffering capacity of diet, health conditions of the flock, as well as environmental factors (Papatsiros et al., 2014; Yang et al., 2018).

The absence of significant changes in carcass characteristics may indicate that the principal effects of OAB were related to feed utilization and intestinal function rather than changes in carcass composition. The present findings are consistent with those of Brzoska et al. (2013) and Nguyen et al. (2018) who reported that dietary supplementation with organic acids did not significantly influence the relative weights of the breast muscle and gizzard, and liver in broiler chickens. Consistent with these findings, Dong et al. (2024) observed no significant differences in the relative weights of the liver, thymus, and bursa of Fabricius by the inclusion of a liquid acidifier in broiler drinking water, which is consistent with our results. However, in contrast to the findings of Dong et al. (2024), no significant effect of OAB supplementation on relative spleen weight was observed in the present study, whereas their liquid acidifier significantly increased this parameter. The lack of change in weights of lymphoid organ in the current study does not imply immunological inactivity, as the observed reduction in the H:L and downregulation of jejunal TNFα expression suggest reduced inflammation and stress.

Intestinal morphology is widely recognized as a key indicator of intestinal development (Li et al., 2022). An increase in VH reflects an expansion of the absorptive surface, which facilitates more efficient nutrient uptake and may ultimately promote better growth performance (Awad et al., 2009; Li et al., 2022; Ravindran & Abdollahi, 2021). In the present study, birds fed OAB showed increased VH and VSA on d 42 and a tendency for improved VSA on d 28, which contributed to their improved growth performance. Previous investigations have likewise demonstrated the beneficial effects of organic acids on intestinal morphology (Adil et al., 2010; Gao et al., 2021; Ghazalah et al., 2011).

The current study demonstrated that OAB supplementation significantly reduced the H:L in broiler chickens. The H:L is a widely accepted and reliable indicator of physiological stress in poultry, with a lower ratio reflecting reduced stress levels (Lee et al., 2022). The observed reduction in H:L ratio is in line with the findings of Dong et al. (2024), who stated improved immune-related indicators in broiler chickens receiving organic acids in drinking water, suggesting a stress-mitigating effect of these additives. Contrary to our results, Akaichi et al. (2022) observed that supplementing broiler diets with 0.02% of an OAB significantly elevated the serum H:L. Furthermore, Abbas et al. (2013) reported that layers provided drinking water supplemented with formic acid exhibited higher antibody titers against NDV than hens receiving unsupplemented water. The observed differences could be explained by variations in the composition of the additives, bird strain, vaccination protocols, sampling time, and experimental conditions.

The present results demonstrate that OAB selectively modulated the jejunal expression of genes involved in antioxidant defense and inflammatory responses. Specifically, OAB significantly upregulated the expression of NFE2L2 and SOD1 while downregulating TNFα, with no significant effects observed on GPX1, NFKB1, IL6, and IL10. The observed increase in NFE2L2 (also known as NRF2) expression is a key finding. NFE2L2 functions as a central transcriptional regulator that controls the expression of a wide range of antioxidant and cytoprotective genes under oxidative stress conditions. On the other hand, maintaining normal NFE2L2 activity is essential for cellular survival, particularly under conditions of elevated oxidative or metabolic stress (Dodson et al., 2019). The upregulation of SOD1, which encodes the superoxide dismutase enzyme responsible for catalyzing the dismutation of superoxide radicals, aligns with the activation of the NFE2L2 pathway (Ma, 2013). This suggests that the OAB enhanced the birds' endogenous antioxidant capacity by stimulating the NFE2L2 signaling cascade, leading to increased expression of downstream antioxidant enzymes like SOD1. A similar finding was reported by Ma et al. (2021), who demonstrated that dietary OAB enhanced antioxidant status by increasing superoxide dismutase activity in the serum of broiler chickens.

TNF-α is a key pleiotropic cytokine involved in the regulation of immune responses, host defense, and the development of both acute and chronic inflammation (Rohde et al., 2018). The significant decrease in TNFα expression in this study indicates that the OAB helped to suppress pro-inflammatory signaling. This finding is particularly relevant as it demonstrates a direct effect on a key inflammatory mediator, which could contribute to improved overall health and reduced systemic inflammation in birds. Consistent with our findings, Qiu et al. (2023) reported lower TNF-α concentrations in the jejunal mucosa of broiler chickens, whereas IL-6 concentrations remained unchanged. In contrast to the present results, Ma et al. (2021) demonstrated that supplementation with an OAB at 6000 mg/kg increased serum IL-10 levels, while serum TNF-α concentrations were not affected.

Serum biochemical indices provide valuable information regarding the health, nutritional, and physiological status of animals (Zálešáková et al., 2025). In the present study, OAB supplementation was associated with higher total protein and albumin concentrations, indicating that OAB might improve the protein synthesis in broiler liver (Ding et al., 2020). In agreement with the present findings, Ding et al. (2020) reported that dietary inclusion of 0.5% fumaric acid increased total protein and albumin levels in broilers. In addition, glucose concentration was increased in OAB-supplemented birds, which is consistent with the findings of Iqbal et al. (2021), who reported elevated serum glucose levels in broiler chickens fed a diet supplemented with the organic acid sodium diformate at 1 g/kg. Contrary to our findings, Adil et al. (2010) reported no significant effect of dietary organic acids on serum glucose concentration in broiler chickens. Consistent with our results, supplementation with organic acids has generally not been shown to affect serum mineral concentrations, including calcium and phosphorus (Akaichi et al., 2022).

The lack of significant differences in the activities of liver enzymes AST, ALT, ALP, and LDH is noteworthy. As the primary metabolic organ in poultry, the liver performs a wide range of essential functions in nutrient metabolism, biosynthesis, digestion, detoxification, and maintenance of physiological homeostasis (Zaefarian et al., 2019). Liver enzymes are widely recognized indicators of liver function and hepatocellular integrity, and elevations in their serum activities generally reflect hepatic injury or cellular leakage (Li et al., 2019). Therefore, the lack of significant alterations in these enzyme activities in OAB-supplemented groups suggests that the organic acid blend did not exert hepatotoxic effects and that liver cell integrity was maintained. This finding is consistent with the results of (Abdel-Fattah et al., 2008), who reported no significant effects of dietary organic acids on serum AST and ALT activities and concluded that organic acids could be included in broiler diets at levels up to 3% without adverse effects on liver or kidney function. In some cases, reductions in ALT and ALP activity have even been observed with certain organic acid combinations, further supporting the safety of these additives (Al-Ghamdi, 2023; Islam et al., 2024).

The most notable findings were the reductions in breast press loss and cooking loss, as well as in thigh press loss, accompanied by a tendency toward lower thigh cooking loss. These responses indicate improved water-holding capacity, which is economically important because water retention influences processing yield, juiciness, and consumer acceptance. In addition, OAB supplementation significantly increased meat pH measured 24 h postmortem, further supporting its beneficial effects on meat quality. Organic acids may improve meat quality through several mechanisms, including enhanced nutrient metabolism, reduced anaerobic glycolytic activity, and improved antioxidant status (Gao et al., 2021). The concurrent upregulation of NFE2L2 and SOD1 in jejunal tissue and the lower H:L ratio may indicate an improved systemic physiological environment. However, because antioxidant markers were not measured in skeletal muscle, these responses cannot be conclusively linked to the observed changes in meat quality. Furthermore, dietary acidifiers may modulate the gut microbiota and its metabolites, which could also contribute to maintaining muscle pH and attenuating its postmortem decline (Gao et al., 2021), a mechanism that is consistent with the higher pH observed in OAB-fed birds. The absence of treatment effects on dry matter, ash, and organic matter contents of breast and thigh muscles suggest that OAB affected specific physical quality characteristics rather than the basic chemical composition of meat. Previous research has likewise shown that organic acids can improve meat quality in broilers (Gao et al., 2021; Lee et al., 2012).

## 5 Conclusion

Dietary supplementation with 0.1% OAB produced age-dependent performance responses in broiler chickens, reducing BWG and FI during the starter period but improving BWG, FCR, and EPEI during the finisher period and FCR over the entire experiment. OAB also enhanced jejunal VH and VSA, reduced the H:L ratio, upregulated jejunal NFE2L2 and SOD1 expression, and downregulated TNFα expression. In addition, OAB improved meat quality. However, these findings should be interpreted with caution because only one commercial OAB formulation at a single inclusion level was evaluated. Further research is warranted to determine whether these responses are consistent across different OAB formulations and inclusion levels.

## Ethics declaration

This study was conducted in accordance with the ARRIVE (Animal Research: Reporting of In Vivo Experiments) guidelines. This study was approved by the Research Animal Ethics Committee of Ilam University, Ilam, Iran. (Approval No. IR.ILAM.REC.1404.019)

## CRediT authorship contribution statement

**Hassan Shirzadi:** Writing – review & editing, Supervision, Software, Methodology, Investigation, Data curation, Conceptualization. **Enayat Rahmatnejad:** Writing – review & editing, Writing – original draft, Methodology, Investigation, Conceptualization. **Hassan Habibi:** Writing – review & editing, Methodology, Investigation.

## Declaration of competing interest

The authors declare that they have no known competing financial interests or personal relationships that could have appeared to influence the work reported in this paper.
