# Supplementary material for: Dietary organic acid supplementation improved productive performance, jejunal morphology, immune status, antioxidant- and inflammation-related gene expression, blood biochemistry, and meat quality of broiler chickens
Source: Vet Anim Sci. 2026 Sep 11;34:100866. doi: 10.1016/j.vas.2026.100866 (PMC13594615; doi:10.1016/j.vas.2026.100866)
Supplement: Supplementary file 1 [file mmc1.docx]

| Day | Temperature (°C) | THI |
| --- | --- | --- |
| 0 | 33 | 30.117 |
| 3 | 31 | 28.427 |
| 6 | 30 | 27.582 |
| 9 | 29 | 26.737 |
| 12 | 28 | 25.892 |
| 15 | 27 | 25.047 |
| 18 | 26 | 24.202 |
| 21 | 25 | 23.357 |
| 24 | 24 | 22.512 |
| 27 | 22 | 20.822 |
| 30 | 22 | 20.822 |
| 33 | 22 | 20.822 |
| 36 | 22 | 20.822 |
| 39 | 22 | 20.822 |
| 42 | 22 | 20.822 |

**Supplementary Figure S1.** Temperature and temperature-humidity index (THI) throughout the 42-day experimental period. From day 27 to day 42, temperature and relative humidity and consequently THI remained constant.
